# Isolation and Assessment of the *in Vitro* Anti-Tumor Activity of Smenothiazole A and B, Chlorinated Thiazole-Containing Peptide/Polyketides from the Caribbean Sponge, *Smenospongia aurea*

**DOI:** 10.3390/md13010444

**Published:** 2015-01-16

**Authors:** Germana Esposito, Roberta Teta, Roberta Miceli, Luca S. Ceccarelli, Gerardo Della Sala, Rosa Camerlingo, Elena Irollo, Alfonso Mangoni, Giuseppe Pirozzi, Valeria Costantino

**Affiliations:** 1 The NeaNat Group, Dipartimento di Farmacia, Università degli Studi di Napoli Federico II, Via D. Montesano 49, 80131 Napoli, Italy; E-Mails: germana.esposito@unina.it (G.E.); roberta.teta@unina.it (R.T.); gerardo.dellasala@unina.it (G.D.S.); alfonso.mangoni@unina.it (A.M.); 2 Department of Experimental Oncology, Istituto Nazionale Tumori Fondazione “G. Pascale”, Via M. Semmola, 80131 Napoli, Italy; E-Mails: r.miceli@istitutotumori.na.it (R.M.); luca.ceccarelli@virgilio.it (L.S.C.); r.camerlingo@istitutotumori.na.it (R.C.); e.irollo@istitutotumori.na.it (E.I.); g.pirozzi@istitutotumori.na.it (G.P.)

**Keywords:** *Smenospongia aurea*, marine natural products, thiazole, structure elucidation, anti-tumor lead molecules, smenamides, solid tumor cell lines, apoptosis, MTT assays

## Abstract

The study of the secondary metabolites contained in the organic extract of Caribbean sponge *Smenospongia aurea* led to the isolation of smenothiazole A (**3**) and B (**4**), hybrid peptide/polyketide compounds. Assays performed using four solid tumor cell lines showed that smenothiazoles exert a potent cytotoxic activity at nanomolar levels, with selectivity over ovarian cancer cells and a pro-apoptotic mechanism.

## 1. Introduction

It is largely recognized that natural products (NPs) are one of the most prolific sources of therapeutics, and because of their enormous structural diversity, they can serve as leads in finding novel drugs to be introduced in cancer therapy. In contrast to the stochastic model, the new concept widely accepted is that tumor cells are heterogeneous, and only a small subset of cancer cells has the ability to initiate new tumors [1]. This subset of cells is also responsible for the invasive growth, dissemination to distant sites and drug resistance. According to this model, these subsets of cells, termed cancer stem cells (CSCs), are biologically and functionally distinct from the bulk of tumor cells and must be specifically targeted by cancer treatments to achieve a permanent cure [2].

In this scenario, there is an emerging need for new chemotherapeutics in order to find new drugs in the fight against cancer and to solve the emergent problem of drug resistance that is responsible for high mortality in cancer patients through initial reduction in effectiveness and subsequent treatment failure. Moreover, novel therapeutic agents are needed for refractory or relapsed patients and for patients with advanced stages of the disease. 

To date, 42% of anticancer drugs used in therapy can be traced back to NPs [3]. Marine invertebrates and bacteria are extraordinarily rich sources of novel molecules. The approval by the EU of trabectedin (Yondelis^®^, PharmaMar, Madrid, Spain) in 2007 as the first marine anticancer drug and of auristatin E, in combination with a monoclonal antibody, in 2011, as a treatment for Hodgkin’s lymphoma [4] gave a new impulse to the research in this field.

**Figure 1:**
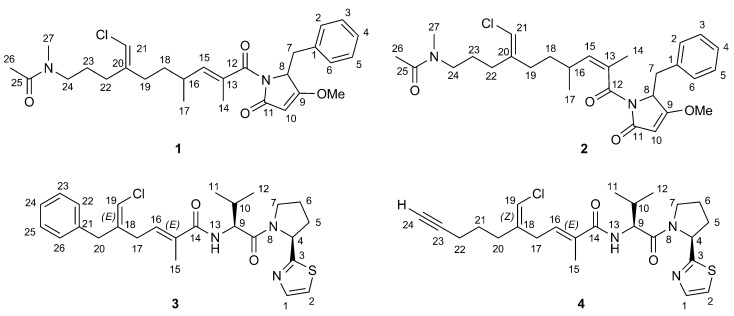
Structures of smenamide A (**1**) and B (**2**) and of smenothiazole A (**3**) and B (**4**).

Our research is focused on the study of the chemistry of natural products from marine sponges to discover novel lead molecules for anticancer [5,6] and anti-inflammatory [7] drug design. Recently, while studying the chemistry of the lipophilic extract of the sponge, *Smenospongia aurea* (order Dictyoceratida, family Thorectidae), collected by scuba along the coast of Little Inagua (Bahamas Islands), we found two novel compounds, smenamide A (**1**) and B (**2**) (Figure 1) [6]. They are hybrid non-ribosomal-peptide/polyketide (NRP/PK) compounds containing a dolapyrrolidinone unit. The structural homology between smenamides and dolastatins, *i.e.*, the dolapyrrolidinone unit, inspired their evaluation as leads in antitumor drug research. Smenamides are indeed potent cytotoxic agents at the nanomolar level on lung cancer Calu-1 cells, their activity being exerted through a clear pro-apoptotic mechanism only for Compound **1**, differing from Compound **2** for the configuration of the C-13 double bond.

Smenamides were isolated in very minute amounts, partly because they were very difficult to separate chromatographically from the large amounts of brominated alkaloids, which are present in *S. aurea*. In an attempt to obtain larger amounts of smenamides, we developed an improved procedure for their isolation. This study revealed the presence of two further chlorinated NRP/PK compounds, smenothiazole A (**3**) and B (**4**), biogenetically related, but structurally very different from smenamides, which exert a potent cytotoxic activity at nanomolar levels, with a selectivity over ovarian cancer cells. Here, we report on the isolation and stereostructural elucidation of smenothiazoles, along with data obtained from MTT assays, cell apoptosis assays and cell cycle analysis performed using four cell lines derived from three different solid tumors. 

## 2. Results

### 2.1. Isolation and Stereostructural Determination

Samples of *S. aurea* collected directly by us along the coast of Little Inagua (Bahamas Islands) were extracted with MeOH/CHCl_3_ mixtures. The organic extract was separated by flash chromatography on RP-18 silica gel. A fraction mostly composed of alkaloids, but also containing smenothiazoles, was partitioned in a two-phase system composed of H_2_O (160 mL), MeOH (260 mL), CHCl_3_ (140 mL) and AcOH (5 mL). Most alkaloids partitioned almost completely into the acidic aqueous phase, while smenothiazoles and smenamides were found in the organic phase. After two subsequent reversed-phase HPLC separations of the latter, pure smenothiazole A (**3**, 225 μg) and B (**4**, 47 μg) were obtained.

The high-resolution ESI mass spectrum of smenothiazole A (**3**) showed an [M + Na]^+^ pseudomolecular ion peak at *m*/*z* 508.1797, along with an M + 2 isotope peak, whose relative intensity (40%) suggested the presence of both a chlorine and a sulfur atom in the molecule. The molecular formula C_26_H_32_ClN_3_O_2_S was in excellent agreement with these data. A full set of homonuclear and heteronuclear two-dimensional NMR spectra (COSY, TOSCY, ROESY, HSQC and HMBC) of **3** was recorded. These spectra allowed for the assignment of all the ^13^C chemical shifts, without the need for recording a 1D ^13^C NMR spectrum.

The proton spectrum was suggestive of an NRP/PK structure, in that it comprised signals for two amino acid α protons at δ 5.47 and 4.54 (the relevant carbons resonating at δ_C_ 60.7 and 58.0, respectively, were identified from HSQC), two olefinic protons at δ 6.16 and 6.12, one methyl singlet at δ 1.79 and two methyl doublets at δ 1.01 and 0.97 and seven aromatic protons. Five of them were attributable to a monosubstituted phenyl ring, while the remaining two were vicinal aromatic protons, whose mutual coupling constant, 3.3 Hz, implied a five-membered heteroaromatic ring [8].

**Figure 2:**
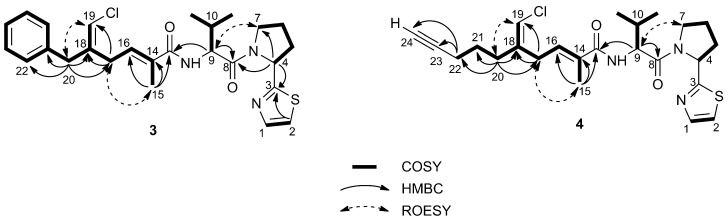
Most significant correlations provided by the COSY, HMBC and ROESY 2D NMR spectra of smenothiazole A (**3**) and B (**4**).

As a result of the presence of several allylic and homoallylic couplings, all of the protons of the polyketide part of the molecule turned out to be part of a single spin system. Thus, the COSY spectrum displayed long-range coupling of: (i) the *ortho* phenyl protons at δ 7.19 (H-22/H-26) with the methylene protons at δ 3.44 (H_2_-20); (ii) the olefinic proton at δ 6.12 (H-19) with H_2_-20 and the methylene protons at δ 3.03 (H_2_-17); and (iii) the methyl protons at δ 1.79 (H_3_-15) with H_2_-17 and the olefinic proton at δ 6.15 (H-16). The structure of the polyketide part of smenothiazole A (**3**) was confirmed by an extensive network of HMBC correlations (Figure 2), which additionally allowed us to assign all of the nonprotonated carbon atoms in this moiety, including the amide carbonyl carbon atom at δ 172.2 (C-13). The presence of a chlorine carbon atom at C-19 was inferred by the chemical shift of C-19 and C-20, both very close to those of the corresponding atoms of smenamide A (**1**) [6].

**Figure 3:**
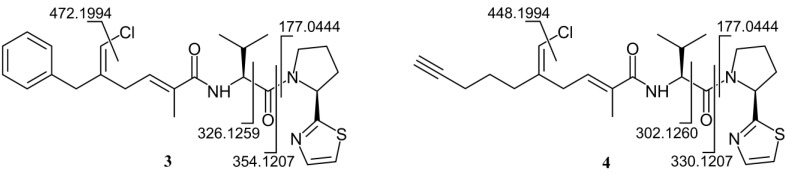
Fragment ions observed in the high-resolution ESI MS/MS spectrum of smenothiazole A (**3**) and B (**4**).

As for the peptide part of the molecule, analysis of the COSY and TOCSY spectra led to the identification of a valine residue (α proton at δ 4.54) and a proline residue (α proton at δ 5.27) (see Table 1 for the assignments). The connection between the Val residue and the polyketide carbonyl group (C-13) was demonstrated by the HMBC correlation of H-9 with C-13 and the connection between Val and Pro residues by HMBC of both H-4 and H-9 with the carbonyl carbon atom at δ 173.5 (C-8). Finally, the remaining atoms required by the molecular formula (1 S, 1 N, 3 C and 2 H) formed a thiazole ring linked to the proline α carbon, as shown by the two doublets at δ 7.71 (H-1) and 7.48 (H-2) in the proton spectrum and the HMBC correlations between H-2 and H-4 with the carbon atom at δ 174.4 (C-3). Finally, a high resolution MS/MS spectrum was recorded using the sodiated pseudomolecular ion peak of **3** (*m*/*z* 508) as the parent ion. All of the observed fragment ions (Figure 3a) were in full agreement with the proposed structure.

**Figure 4:**
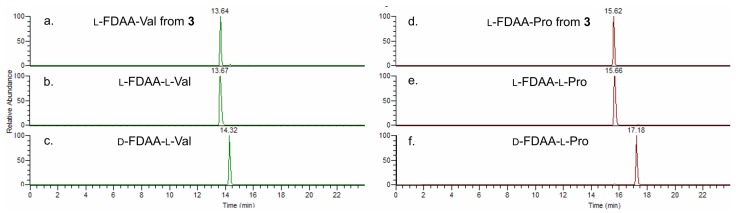
HR-ESI-MS-HPLC analysis of Marfey’s derivatives from smenothiazole A (**3**): extracted-ion chromatograms at *m*/*z* 370.1357 of l-1-fluoro-2-4-dinitrophenyl-5-alanine amide (FDAA)-Val from **3** (**a**); of authentic l-FDAA-l-Val (**b**); and of authentic d-FDAA-l-Val (**c**); extracted-ion chromatograms at *m*/*z* 368.1201 of l-FDAA-Pro from **3** (**d**); of authentic l-FDAA-l-Pro (**e**); and of authentic d-FDAA-l-Pro (**f**).

The configuration of the two double bonds was established from the NOESY spectrum. Specifically, the 14*E* and 18*E* configurations were based, respectively, on the NOESY correlations between H-19 and H_2_-20 and between H_3_-15 and H_2_-17. Marfey’s analysis [9] was used to determine the configuration of the two amino acids. Because of the propensity for racemization at the α carbon of thiazole-modified amino acid [10], Compound **3** was subjected to ozonolysis prior to hydrolysis and derivatization with the l-enantiomer of Marfey’s reagent (FDAA, or 1-fluoro-2-4-dinitrophenyl-5-alanine amide; see the Experimental Section for details). Only 4 μg of Compound **3** were subjected to degradation, and the obtained derivatives were analyzed by high resolution ESIMS-HPLC to make the analysis as sensitive and specific as possible. In spite of the low amounts used, the extracted-ion chromatograms at *m*/*z* 370.1357 (FDAA-Val) and 368.1201 (FDAA-Pro) were almost devoid of noise (Figure 4). Both amino acids were found to have the l configuration on the basis of the retention times of their respective Marfey’s derivatives.

**Table 1:** NMR data of smenothiazole A (**3**) (700 MHz, CD_3_OD).

Pos.		δ_H_ [mult., *J* (Hz)]	δ_C_ [mult.]	COSY	HMBC
1		7.71 (d, 3.3)	142.9 (CH)	2	
2		7.48 (d, 3.3)	120.1 (CH)	1	1, 3
3		-	174.4 (C)		
4		5.47 (dd, 8.2, 2.9)	60.7 (CH)	5a, 5b	3, 5, 6, 7, 8
5	a	2.37 (m)	33.2 (CH_2_)	4, 5b, 6a, 6b	3, 4, 6, 7
	b	2.21 (m)		4, 5a, 6a, 6b	3, 6
6	a	2.17 (m)	25.4 (CH_2_)	4a, 5b, 6b, 7a, 7b	3, 7
	b	2.11 (m)		4a, 5b, 6a, 7a, 7b	4, 5, 7
7	a	4.01 (ddd, 10.1, 8.4, 7.1)	48.7 (CH_2_)	6a, 6b, 7b	5, 6
	b	3.88 (ddd, 10.1, 7.8, 4.0)		6a, 6b, 7a	5, 6
8		-	173.5 (C)		
9		4.54 (d, 7.9)	58.0 (CH)	10	8, 10, 11, 12, 13
10		2.14 (m)	31.9 (CH)	9, 11, 12	8, 9, 11, 12
11		1.01 (d, 6.7)	19.9 (CH_3_)	10	9, 10, 12
12		0.97 (d, 6.7)	18.9 (CH_3_)	10	9, 10, 11
13		-	172.2 (C)		
14		-	133.4 (C)		
15		1.79 (br. t, 1.5)	13.2 (CH_3_)	16, 17	13, 14, 16
16		6.16 (tq, 7.5, 1.5)	133.4 (CH)	15, 17	14, 15, 17
17		3.03 (br. d, 7.5)	30.2 (CH_2_)	15, 16, 19	14, 16, 18, 19, 20
18		-	141.3 (C)		
19		6.12 (br. s)	116.0 (CH)	17, 20	17, 18, 20
20		3.44 (br. s)	41.9 (CH_2_)	19, 22/26	17, 18, 19, 21, 22/26
21		-	139.6 (C)		
22/26		7.19 (br. d, 8.2)	129.9 (CH)	19, 23/25, 24	20, 24, 26/22
23/25		7.29 (br. t, 8.2)	129.4 (CH)	22/26, 24	21, 25/23
24		7.21 (br. t, 8.2)	128.1 (CH)	22/26, 23/25	22/26

The molecular formula of smenothiazole B (**4**) was determined as C_24_H_32_ClN_3_O_2_S from the [M + Na]^+^ pseudomolecular ion peak at *m*/*z* 484.1786 in the high resolution ESI mass spectrum and only differed from smenothiazole A by two less carbon atoms, thus implying two less unsaturations. The proton NMR spectrum of Compound **4** was similar to that of **3**, and most signals displayed a similar chemical shift and identical multiplicity (Table S1).

However, the signals of aromatic rings and of the benzylic methylene were absent, suggesting that the difference between the two molecules was located in their western moiety. Two ^13^C signals at δ 71.0 (CH) and 84.4 (C) suggested a terminal alkyne. The one-bond correlation between the alkyne proton (H-24, δ 2.25) and the relevant carbon was not detectable in the standard HSQC spectrum (optimized for ^1^*J*_CH_ = 150 Hz) because of the large ^1^*J*_CH_ coupling constant characteristic of terminal alkynes, but was clearly visible in an HSQC experiment optimized for ^1^*J*_CH_ = 250 Hz. The alkyne proton H-24 displayed a 2.8-Hz long-range coupling with the methylene group at δ 2.18 (H_2_-22), which was, in turn, connected through two additional methylene groups to the vinyl chloride group, as shown by the COSY spectrum. Comparison of NMR data showed that the rest of the molecule is the same as Compound **3**, and this was confirmed by the fragments observed in the high-resolution ESI MS/MS spectrum (Figure 3).

The configuration of the two double bonds is also the same as Compound **3** (NOESY correlation peaks are present between H-19 and H_2_-20 and between H_3_-15 and H_2_-17). It should be noted, however, that in Compound **4**, the priority of the groups linked to C-18 is different, so that the relevant double-bond configuration is denoted as 18*Z* for **4** and 18*E* for **3**. Finally, Marfey’s analysis as described above was used to determine the l configuration of the Pro and Val residues.

### 2.2. In Vitro Evaluation of Cytotoxic Activity of Smenothiazoles

#### 2.2.1. Morphological Changes Induced by Treatment with Smenothiazoles Lead to Apoptosis

Peptide/polyketide hybrid natural products of cyanobacterial origin often show cytotoxic activity to cancer cells, which range from moderate to extraordinarily high, as in the case of dolastatin-10 and jamaicamides. The structural analogy of smenothiazole B (**3**) with the western part of jamaicamide B and the biosynthetic relationship of both smenothiazoles with the cytotoxic smenamides 1 and 2 suggested that the cytotoxic activity be explored further.

In order to investigate the effect of smenothiazoles on different solid tumors, four cell lines derived from three different solid tumors (Calu-1 and LC31 lung-cancer cells, A2780 ovarian cancer cells and MCF7 breast cancer cells) were treated for 48 h with different concentrations of the two compounds. After this time, the cells appeared elongated and flattened under the optical microscope, and some showed small vacuoles in their cytoplasm, indicative of cell death. These morphological changes were observed at concentrations of 50 nM and above. Moreover, at 100 nM, all cells were in suspension, suggesting that they were dead. In contrast, the morphology of treated cells was similar to that of untreated cells at concentrations of 1, 10 and 30 nM.

#### 2.2.2. Smenothiazoles Reduce Cell Viability

**Figure 5:**
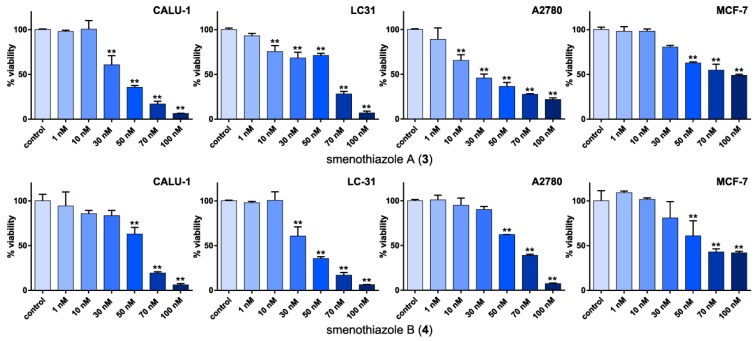
Evaluation by the MTT assay of viability of four cancer cell line after 48 h of treatment with smenothiazole A (**3**) and smenothiazole B (**4**). ** *p* < 0.0005.

In order to investigate the *in vitro* activity of smenothiazoles, MTT assays were performed (Figure 5). The MTT assay is a simple way to measure cell viability and is the first step for testing the cytotoxic effect of a compound. Viable cells with active metabolism convert MTT (3-(4,5-dimethylthiazol-2-yl)-2,5-diphenyltetrazolium bromide) into the corresponding, purple-colored formazan, while dead cells lose their ability to convert MTT into formazan.

Smenothiazoles significantly reduced cell viability in the Calu-1 and LC31 lung-cancer cell lines and in the A2780 ovary cancer cell lines. Some differences were observed among different cell lines, as well as between the two compounds, but no general trend could be drawn. It is noteworthy, however, that smenothiazole A (**3**) was significantly active on the A2780 ovarian cells at only 10 nM. The activity on the breast-cancer MCF-7 cells was less remarkable, and cell viability was still in the range of 40%–50% of the control, even at the highest concentration tested.

The presented data clearly show that smenothiazoles are active on tumor cell lines in the low nanomolar range, and they show some selectivity.

#### 2.2.3. Smenothiazoles Induce Apoptosis with Different Percentage

To evaluate whether the cytotoxic activity was related to apoptosis induction, Annexin-V FITC/PI assays were performed. Annexin binds to phosphatidylserine when it is exposed on the outer leaflet of the plasma membrane, and PI binds to DNA only when the cells is dead. Viable cells exclude PI and are negative for the bond with annexin. Cells in early apoptosis are positive for annexin, but negative for PI. Cells that are positive for both annexin and PI are cells in late apoptosis, whereas necrotic cells are positive only for PI and negative for annexin. Therefore, this method allows live cells to be distinguished from those in apoptosis and those with necrosis.

**Figure 6:**
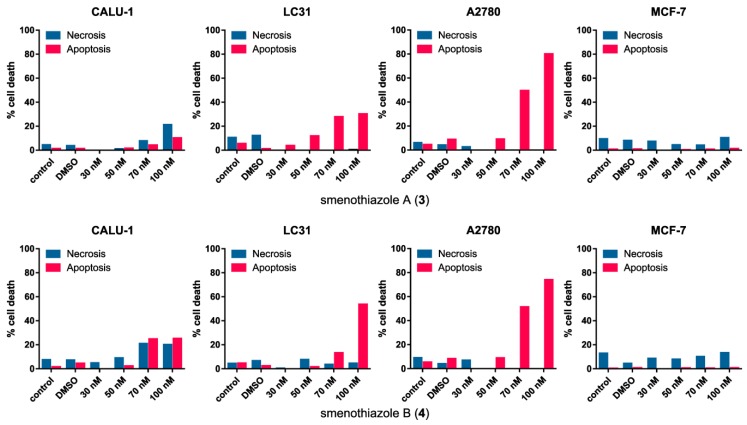
Evaluation assays of apoptosis/necrosis by the Annexin-V FITC/PI in four cancer cell lines after 48 h of treatment with smenothiazole A (**3**) and smenothiazole B (**4**).

Smenothiazoles were assayed on the same four cell lines used for MTT assays. The results are reported in Figure 6. For Calu-1 cells, the efficacy of smenothiazoles in these assays was limited, and most cells remained viable even at the highest concentration tested. Smenothiazol A (**3**) showed mostly a necrotic effect, while the effect of smenothiazol B (**4**) was borderline, with a percentage of necrotic cells similar to that of apoptotic cells. Better activity was observed on the LC31 primary lung cancer cell line: both compounds showed a clear pro-apoptotic activity, although the slope of the dose-response curve appeared to be different for the two compounds. No effect, either apoptotic or necrotic, was observed for breast MCF-7 cells compared to the control.

**Figure 7:**
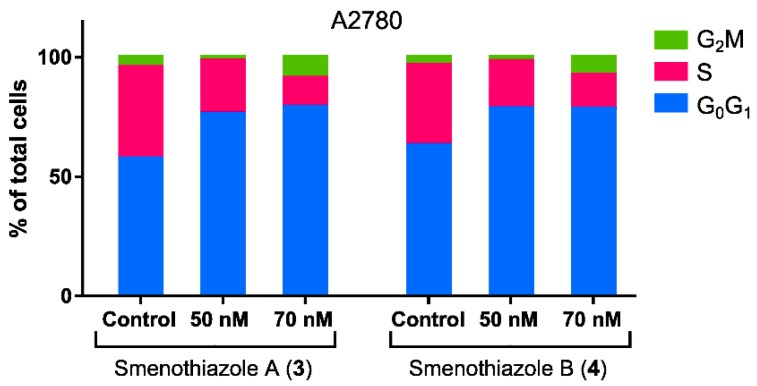
Cell-cycle analysis by flow cytometry of A2780 ovarian carcinoma cells after 48 h of treatment with smenothiazoles.

The most significant activity was observed on the A2780 ovarian carcinoma line. Both compounds were clearly able to induce apoptosis on this line at 70 and 100 nM. In order to evaluate whether these compounds can affect the phases of the cell cycle, untreated and treated A2780 cells were stained with propidium iodide. Analysis of the cell cycle showed that both compounds are able to reduce the number of cells in the S phase at 50 and 70 nM (Figure 7), leading to a block in the G_0_G_1_ phase.

## 3. Discussion

Our drug discovery program focused on novel leads in anticancer drug discovery led to the isolation of two bioactive natural products, smenothiazole A (**3**) and B (**4**). The structure of smenothiazoles was determined by NMR and mass spectroscopy, while elucidation of the configuration of the amino acid components was achieved with only 4 μg of each compound using ozonolysis followed by Marfey’s analysis.

**Figure 8:**
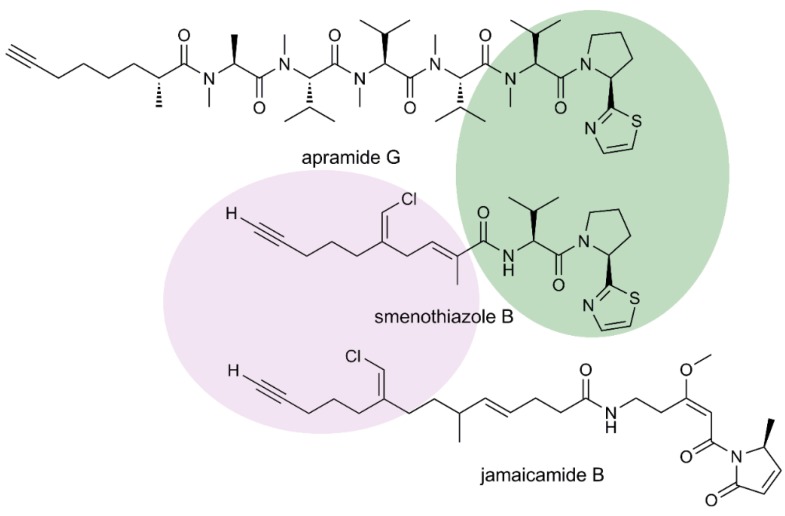
Common structural features of smenothiazole B, apramide G and jamaicamide B.

Although structurally new, smenothiazoles contain structural motifs that are present in various cyanobacterial metabolites. The eastern part of both smenothiazoles is very similar to that of apramide G [11]. The western part of smenothiazole B (**4**) closely resembles that of jamaicamide B [12], a peptide/polyketide belonging to a novel class of marine neurotoxins that shares with smenothiazole B the vinyl chloride moiety and the alkynyl terminus (Figure 8). Both metabolites are produced by the marine cyanobacteria, *Lyngbya majuscula* (recently assigned to the novel genus, *Moorea*, and renamed as *Moorea producens* [13]), a species extremely intriguing from a drug discovery perspective. In contrast, no close analogue exists of the western part of smenothiazole A (**3**).

Interestingly, while smenothiazole B and jamaicamide B share the same vinyl chloride moiety, they show the opposite configuration at the double bond. The pendant vinyl chloride moiety in smenamides and smenothiazoles is very likely to be formed through a unique mechanistic transformation involving an HCS-like (3-hydroxy-3-methylglutaryl-CoA synthase-like)-containing gene cassette. Such biosynthetic machinery is responsible for the β-branching of the polyketide scaffold, generating an extraordinary chemical diversity in marine natural products from cyanobacteria. Two thoroughly studied examples of cyanobacterial HCS transformations are observed in the biosynthesis of curacin A and jamaicamides [14], where the HCS-derived pendant methyl group is converted into a cyclopropane ring in the curacin A pathway and in a vinyl chloride group in the jamaicamide pathway, respectively.

The many structural features in common with cyanobacterial metabolites strongly suggest that smenothiazoles are a product of cyanobacterial metabolism. We have previously found that *S. spongiarum* is the only cyanobacterium present in *S. aurea* [6], and further studies are in progress to prove that it is the real producer of smenamides and smenothiazoles. Two modular biosynthetic systems are used to build these metabolites, namely nonribosomal peptide synthases (NRPSs), which assemble amino acids, and polyketides synthases (PKSs), which link together acetate building blocks. This mixed biosynthetic machine, which also includes further steps, such as oxidations, halogenations and methylations, may produce a truly extraordinary number of secondary metabolites of great value in bio-sustainable drug discovery. It is worth mentioning in this regard that the first example of the production of an NRPS-PKS metabolite (4-*O*-demethylbarbamide, a potent molluscicide) in a heterologous host has been recently published [15]. Heterologous gene expression technologies will indeed play a crucial and fundamental role in drug discovery program and bio-sustainable large-scale production. So far, PCR screening of PKS systems from the metagenomic DNA of the marine sponge *S. aurea* has revealed the presence of the *swf* gene cluster, a new group of widespread mono-modular type-I PKS/FAS specifically associated with sponge symbionts [16]. However, the isolation of peptide/polyketide hybrids from *S. aurea* strongly suggests the existence of unexplored PKS/NRPS pathways in *S. aurea*, which deserve to be extensively investigated to exploit in depth its biosynthetic potential.

Smenothiazoles were tested on four cell lines derived from three different solid tumors, including lung, ovarian and breast cancers. Smenothiazoles exert a potent cytotoxic activity in a dose-dependent manner at low nanomolar levels, with a selectivity on ovarian cancer cells. MTT assays showed comparable activity on Calu-1, LC31 and A2780 cell lines, while the effect on MCF7 cells was lower. However, when tested using Annexin-V FITC/PI assays, smenothiazoles showed a clear selectivity in their ability to induce apoptosis. A strong apoptotic effect was observed with the ovarian cancer cells, A2780, a lesser one with the LC31 cells, while little or no effect was observed with the Calu-1 and MCF7 cells at the doses tested. In addition, the apoptotic effect on A2780 cells is associated with a decrease of the S phase and to the block of the G_0_G_1_ phase of the cellular cell cycle.

Overall, the results reported above show that smenothiazoles have a different behavior on different solid tumors, with a selectivity over the ovarian cancer cells, A2780. No interesting differences associated with the structural features of smenothiazol A and B were observed. Therefore, the western part of the molecule does not seem to affect the activity. Further studies are planned to reveal the molecular mechanism of the signal transduction pathways of this new class of compounds.

## 4. Experimental Section

### 4.1. General Experimental Procedures

High-resolution ESI-MS and HR-ESI-HPLC experiments were performed on a Thermo LTQ Orbitrap XL mass spectrometer (Thermo Fisher Scientific Spa, Rodano, Italy) coupled to an Agilent model 1100 LC system (Agilent Technology, Cernusco sul Naviglio, Italy). The spectra were recorded by infusion into the ESI source using MeOH as the solvent. CD spectra were recorded on a Jasco J-710 spectrophotometer (Easton, MD, USA) using a 1-cm cell. NMR spectra were determined on Varian Unity Inova spectrometers (Agilent Technology, Cernusco sul Naviglio, Italy) at 700 MHz; chemical shifts were referenced to the residual solvent signal (CD_3_OD: δ_H_ 3.31, δ_C_ 49.00). For an accurate measurement of the coupling constants, the one-dimensional ^1^H NMR spectra were transformed at 64-K points (digital resolution: 0.09 Hz). Through-space ^1^H connectivities were evidenced using a ROESY experiment with a mixing time of 450 ms. The HSQC spectra were optimized for ^1^*J*_CH_ = 142 Hz and the HMBC experiments for ^2,3^*J*_CH_ = 8.3 Hz. High performance liquid chromatography (HPLC) separations were achieved on a Agilent 1260 Infinity Quaternary LC apparatus (Agilent Technology, Cernusco sul Naviglio, Italy) equipped with adiode-array detector (DAD).

### 4.2. Collection, Extraction and Isolation

A specimen of *Smenospongia aurea* was collected by scuba along the coast of Little Inagua (Bahamas Islands) during the 2013 Pawlik expedition. The collection was made by specialists that are able to collect selectively the sample needed in small amounts and without affecting the organism. After collection, the sample was immediately identified onboard following the information reported on the website, The Sponge Guide [17]. The sample was frozen immediately after collection and stored at −20 °C until extraction. The sponge (712 g wet weight) was homogenized and extracted with MeOH (4 × 4 L), MeOH and CHCl_3_ in different ratios (2:1, 1:1, 1:2) and then with CHCl_3_ (2 × 4 L). The MeOH extracts were partitioned between H_2_O and *n*-BuOH; the BuOH layer was combined with the CHCl_3_ extracts and concentrated *in vacuo*.

The total organic extract (16.31 g) was chromatographed on a column packed with RP-18 silica gel. The fraction that was eluted with MeOH/H_2_O (9:1) (363.7 mg) was partitioned in a two-phase system composed of H_2_O (160 mL), MeOH (260 mL), CHCl_3_ (140 mL) and AcOH (5 mL); the organic layer, containing smenothiazoles and smenamides, was subjected to reversed-phase HPLC separation (column 250 × 10 mm, 10 μm, Luna (Phenomenex) C18; Eluent A: H_2_O; Eluent B: MeOH; gradient: 55%→100% B over 60 min, flow rate 5 mL/min), thus affording a fraction (*t*_R_ = 32 min) containing Compound **3** and a fraction (*t*_R_ = 28 min) containing Compound **4**. The two fractions were each separated on reversed-phase HPLC (column 250 × 4.6 mm, 5 μm, Luna (Phenomenex) C18; Eluent A: H_2_O; Eluent B: ACN; gradient: 50%→100% B, over 35 min, flow rate 1 mL·min^−1^), which gave 225 µg of pure Compound **3** (*t*_R_ = 20 min) and 47 μg of pure Compound **4** (*t*_R_ = 16 min).

### 4.3. Absolute Configuration of Amino Acids

#### 4.3.1. Ozonolysis and Hydrolysis

A small amount of Compound **3** (4 μg) and **4** (3 μg) were separately suspended in ozone-saturated MeOH (300 μL) at −78 °C for 5 min. The samples were dried under a N_2_ stream to remove the ozone, then treated with 6 N HCl and heated in a flame-sealed glass tube at 180 °C for 2 h. The residual HCl fumes were removed *in vacuo*.

#### 4.3.2. Marfey’s Derivatization with d- and l-FDAA

The hydrolysate of **3** was dissolved in triethylamine/ACN (2:3) (80 µL), and this solution was then treated with 1% 1-fluoro-2,4-dinitrophenyl-5-l-alaninamide (L-FDAA) in ACN/acetone (1:2) (75 μL). The vial was heated at 50 °C for 1 h. The mixture was dried and resuspended in ACN/H_2_O (5:95) (500 μL) for subsequent LC-MS analysis (I). The hydrolysate of **4** was treated with d-FDAA under identical conditions as **3** (II). Authentic l-Val and l-Pro standards were treated with l-FDAA and d-FDAA as above (III).

#### 4.3.3. LC-MS Analysis

The Marfey’s derivatives were analyzed by HR-ESI-MS-HPLC. A 5-μm Kinetex C18 column (100 × 2.10 mm), maintained at room temperature, was eluted at 250 μL·min^−1^ with H_2_O and ACN, using a gradient elution. The gradient program was as follows: 5% ACN 3 min, 5%–60% ACN over 20 min, 90% ACN 5 min. Mass spectra were acquired in positive ion detection mode, and the data were analyzed using the suite of programs, Xcalibur. The retention times of the Marfey’s derivatives (III) and (I) were the following *t*_R_ = (d/l) in min: valine (*m*/*z* 370.1357, [M + H]^+^) *t*_R_ = 14.32/13.67; proline (*m*/*z* 368.1201, [M + H]^+^) *t*_R_ = 17.18/15.66; l-FDAA-Val from **3** *t*_R_ = 13.64; l-FDAA-Pro from **3** *t*_R_ = 15.62. In the same conditions, the retention times for the Marfey’s derivatives (III) and (II) in min *t*_R_ = (d/l) were: valine *t*_R_ = 11.52/9.25; proline *t*_R_ = 9.64/7.31; d-FDAA-Val from **4** *t*_R_ = 11.91; d-FDAA-Pro from **4** *t*_R_ = 9.82.

### 4.4. Smenothiazole A (**3**)

Colorless amorphous solid, HRESIMS (positive ion mode, MeOH) *m*/*z* 508.1797 ([M + Na]^+^, C_26_H_32_ClN_3_NaO_2_S^+^, calcd. 508.17946); MS isotope pattern: M (100%), M + 1 (31%, calcd. 31%), M + 2 (40%, calcd. 41%), M + 3 (11%, calcd. 13%); HRESIMS/MS (parent ion *m*/*z* 508.18 C_26_H_32_ClN_3_NaO_2_S^+^): *m*/*z* 472.1994 (C_26_H_31_O_2_N_3_NaS^+^, calcd. 472.2029), *m*/*z* 354.1207 (C_19_H_22_O_2_NClNa^+^, calcd. 354.1231), *m*/*z* 326.1259 (C_18_H_22_ONClNa^+^, calcd. 326.1282), *m*/*z* 177.0444 (C_7_H_10_N_2_NaS^+^, calcd. 177.0457). ^1^H and ^13^C NMR: Table 1. UV (MeOH): λ_max_ (ε) 357 nm (780) 295 nm (3500), 241 nm (19,400); CD (MeOH): λ_max_ (Δε) 234 (–12.5).

### 4.5. Smenothiazole B (**4**)

Colorless amorphous solid, HRESIMS (positive ion mode, MeOH) *m*/*z* 484.1786 ([M + Na]^+^, calcd. C_24_H_32_ClN_3_NaO_2_S^+^ 484.1796); MS isotope pattern: M (100%), M + 1 (28%, calcd. 28%), M + 2 (41%, calcd. 41%), M + 3 (10%, calcd. 11%); HRESIMS/MS (parent ion *m*/*z* 484.18, C_24_H_32_ClN_3_NaO_2_S^+^): *m*/*z* 448.1994 (C_24_H_31_O_2_N_3_NaS^+^, calcd. 448.2029), *m*/*z* 330.1207 (C_17_H_22_O_2_ClNNa^+^, calcd. 330.1231), *m*/*z* 302.1260 (C_16_H_22_OClNNa^+^, calcd. 302.1282), *m*/*z* 177.0444 (C_7_H_10_N_2_NaS^+^, calcd. 177.0457). ^1^H and ^13^C NMR: Supplementary Table S1; UV (MeOH): λ_max_ (ε): 287 nm (13,000), 246 nm (40,600), 231 nm (63,700); CD (MeOH): λ_max_ (Δε): 234 (–31.6).

### 4.6. Cell Culture

LC31 cell line was obtained in our laboratory by a patient affected from lung squamous adenocarcinoma with written informed consent, approved by the Internal Ethical Committee (National Cancer Institute, Naples, Italy). Calu-1 and MCF-7 cell lines was obtained from the American Type Culture Collection (ATCC). LC31, Calu-1 and MCF7 were cultured in IMDM (Lonza) at 10% FBS, 100 units/mL penicillin plus 100 µg/mL streptomycin (Lonza, Basel, Switzerland) and 250 mg/mL amphotericin B (Lonza, Basel, Switzerland). The cells were treated with different concentrations of smenothiazol A and B for 48 h. Smenothiazol A and B were prepared by diluting the powder with DMSO. For experiments, cells were grown to 70%–80% confluence.

A2780 were generously provided by Eugenio Erba (Flow Cytometry Unit, Mario Negri Institute). Cells were cultured in RPMI 1640 (Lonza, Milan, Italy) supplemented with 10% fetal bovine serum (FBS; Biochrom, Berlin, Germany), 2 mM l-glutamine (PAA Laboratories), 100 units/mL penicillin plus 100 μg/mL streptomycin (Lonza, Basel, Switzerland), 250 mg/mL amphotericin B (Lonza, Basel, Switzerland) at 37 °C, 5% CO_2_.

### 4.7. MTT Assay

Cell viability was determined by the 3-(4,5-dimethylthiazol-2-yl)-2,5-diphenyltetrazolium bromide (MTT) assay according to the manufacturer’s instructions. Briefly, cells were plated in 96-well plates, at a density of 3 × 10^3^ cells/well, in a total volume of 100 μL of complete medium. After treatment for 48 h with smenothiazol A and B at different concentrations (1, 10, 30, 50, 70 and 100 nM), MTT (5 mg/mL in PBS) was added to each well, and the cells were incubated for 4 h at 37 °C. Dimethylsulfoxide (DMSO) was added to each well after removal of the supernatant, and the plates were shaken to dissolve the formazan. The absorbance reading of each well was determined using a computer-controlled microtiter plate reader (Bio-Rad, Hercules, CA, USA) at a wavelength of 540 nm. All experiments were performed in triplicate for each condition. 

The optical density of the cells incubated with culture medium alone was taken as 100% viability. Differences between groups were determined by analysis of variance (ANOVA) and were considered statistically significant at *p* < 0.005. 

### 4.8. Cell Apoptosis Assay

Apoptosis was assessed using the Annexin-V FITC kit (Milteny Biotec, Bergisch Gladbach, Germany) according to the manufacturer’s instructions. Briefly, cells were plated in 6-well plates at a density of 1 × 10^5^ cells/well and were treated for 48 h with smenothiazol A and B at concentrations of 30, 50, 70 and 100 nM.

After treatment cells were recovered, washed with binding buffer (BB) and centrifuged. Annexin-V FITC (10 μL) was added to the pellets, which were then incubated in the dark for 15 min at room temperature. Next, cells were washed and re-suspended in BB, and propidium iodide (PI) solution (20 μg/mL) was added immediately prior to analysis by flow cytometry. The flow-cytometric analysis was performed using a BD FACS ARIA II, and the data were analyzed by Kaluza software. 

### 4.9. Cell Cycle Analysis

Human cell lines, LC31, Calu-1, MCF-7 and A2780, were seeded in 6-well plates. After attaining confluency, they were treated for 48 h with smenothiazol A and B at 50 and 70 nM. After treatment, cells were recovered, washed with phosphate-buffered saline (PBS), centrifuged and the supernatant removed, then 70% ethanol was added to fix cells that were kept at −20 °C for at least 20 min. Afterwards, cells were centrifuged, washed twice with PBS and stained with a solution of RNase (1 mg/mL) and propidium iodide (PI) (2.5–5 μg/mL). Cells were incubated at 4 °C overnight. Cytometric analysis was performed using a BD FACS ARIA II (Berton & Dickinson, Mountain View, CA, USA), and the data were analyzed by the Modfit software (Topsham, ME, USA).

### 4.10. Statistical Analysis

Statistical analysis was done with GraphPad Prism version 6.0 software (GraphPad software Inc., La Jolla, CA, USA).

## 5. Conclusions

The study of the organic extract of the Caribbean sponge *Smenospongia aurea* led to the isolation of smenothiazole A (3) and B (4), unique hybrid peptide/polyketide secondary metabolites. Analyses of their possible role as lead compounds in anticancer drug research was started performing cytotoxic assays using four solid tumor cell lines. Data obtained showed that smenothiazoles exert a potent cytotoxic activity at nanomolar levels, with selectivity over ovarian cancer cells and a pro-apoptotic mechanism.

## Supplementary Files

Supplementary File 1
