# Supplementary material for: Isolation and Assessment of the in Vitro Anti-Tumor Activity of Smenothiazole A and B, Chlorinated Thiazole-Containing Peptide/Polyketides from the Caribbean Sponge, Smenospongia aurea
Source: Mar Drugs. 2015 Jan 16;13(1):444–59. doi: 10.3390/md13010444 (PMC4306946; doi:10.3390/md13010444)
Supplement: Supplementary File 1 [file marinedrugs-13-00444-s001.pdf]

Supplementary Information

Table S1. NMR data of smenothiazole B (**4**) (700 MHz, CD_3OD).

**Pos.**		**δ_H [mult., *J* (Hz)]**	**δ_C [mult.]**	**COSY**	**HMBC**
1		7.70 (d, 3.3)	143.1(CH)	2, 4	
2		7.47 (d, 3.3)	120.4 (CH)	1, 4	
3		-	174.4 (C)		
4		5.46 (dd, 8.2, 2.9)	60.5 (CH)	1, 2, 5a, 5b	
5	a	2.35 (m)	33.2 (CH_2)	4, 5b, 6a, 6b	
	b	2.20 (m)		4, 5a, 6a, 6b	
6	a	2.15 (m).	25.4 (CH_2)	4a, 5b, 6b, 7a, 7b	
	b	2.11 (m)		4a, 5b, 6a, 7a, 7b	
7	a	4.02 (ddd, 10.1, 8.4, 7.1)	48.9 (CH_2)	6a, 6b, 7b	
	b	3.88 (ddd, 10.1, 7.8, 4.0)		6a, 6b, 7a	
8		-	173.5 (C)	-	
9		4.55 (d, 7.9)	58.3 (CH)	10	8, 10, 11, 13
10		2.16 (m)	31.9 (CH)	9, 11, 12	8, 9, 11, 12
11		1.01 (d, 6.7)	19.9 (CH_3)	10	9, 10, 12
12		0.98 (d, 6.7)	18.9 (CH_3)	10	9, 10, 11
13		-	172.1 (C)	-	
14		-	133.3 (C)	-	
15		1.94 (br. t, 1.5)	13.3 (CH_3)	16, 17	13, 14, 16
16		6.27 (tq, 7.5, 1.5)	133.6 (CH)	15, 17	15
17		3.14 (br. d, 7.5)	30.3 (CH_2)	15, 16, 19	14, 16, 18, 19, 20
18		-	141.3 (C)	-	
19		6.04 (br. s)	115.2 (CH)	17, 20	17, 18, 20
20		2.25 (br. t, 7.7)	34.6 (CH_2)	19, 21	17, 18, 19, 21, 22
21		1.65 (quintet, 7.3)	27.6 (CH_2)	20, 22	18, 20, 22, 23
22		2.18 (ddd, 7.0, 7.0, 2.8)	18.5 (CH_2)	21, 24	20, 21, 23, 24
23		-	84.4 (C)	-	
24		2.25 (t, 2.8)	71.0 (CH)	22	

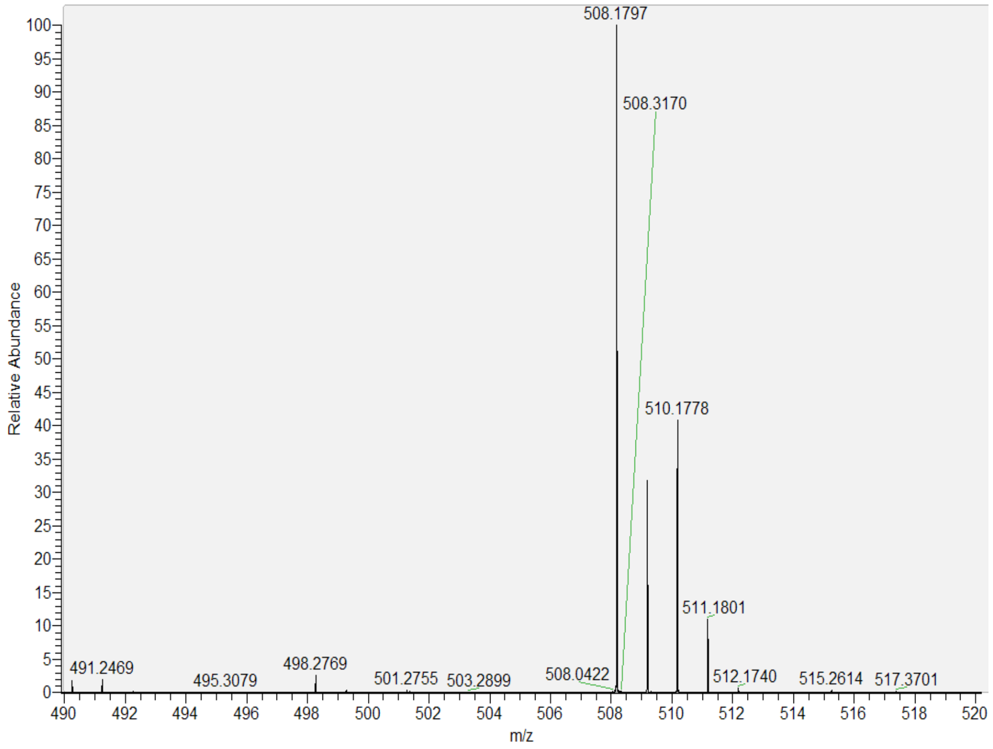

Figure S1. Positive ion mode high-resolution ESI MS spectrum of smenothiazole A (**3**).

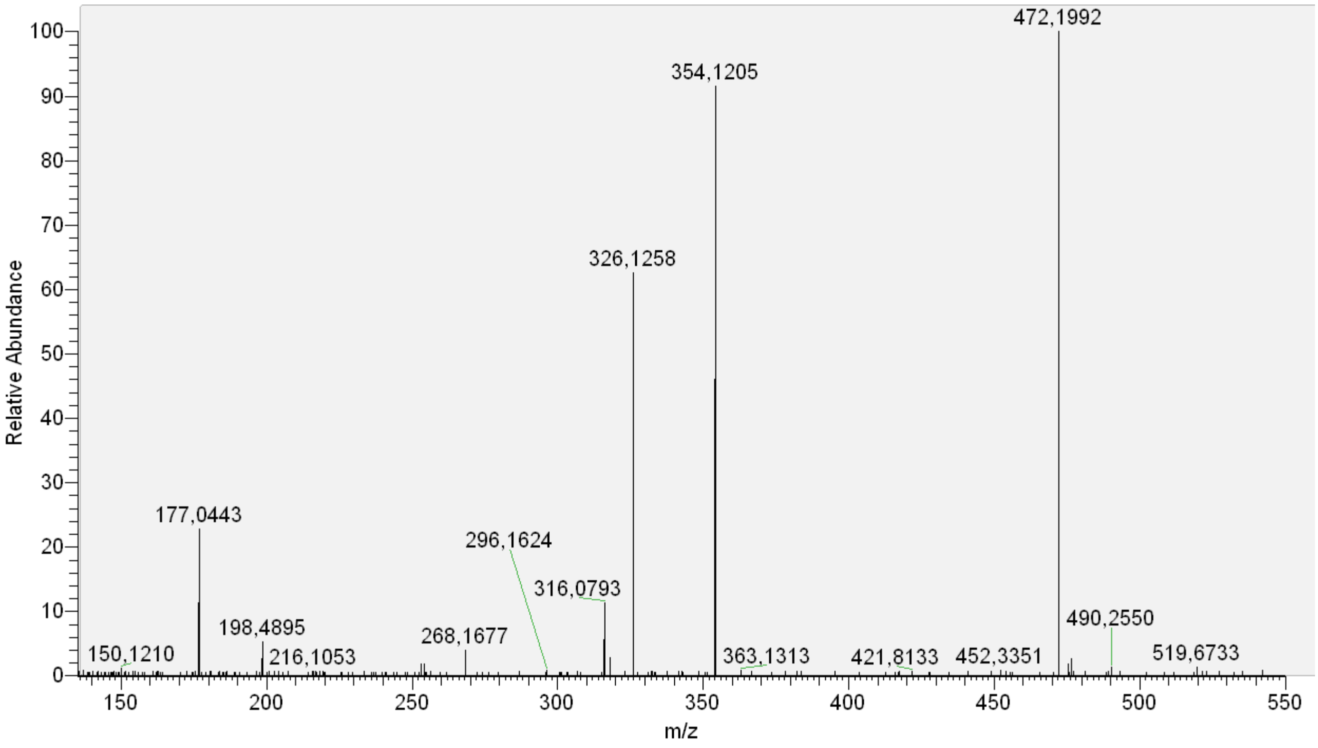

Figure S2. Positive ion mode high-resolution ESI MS/MS spectrum of smenothiazole A (**3**), parent ion at *m*/*z* 508.18.

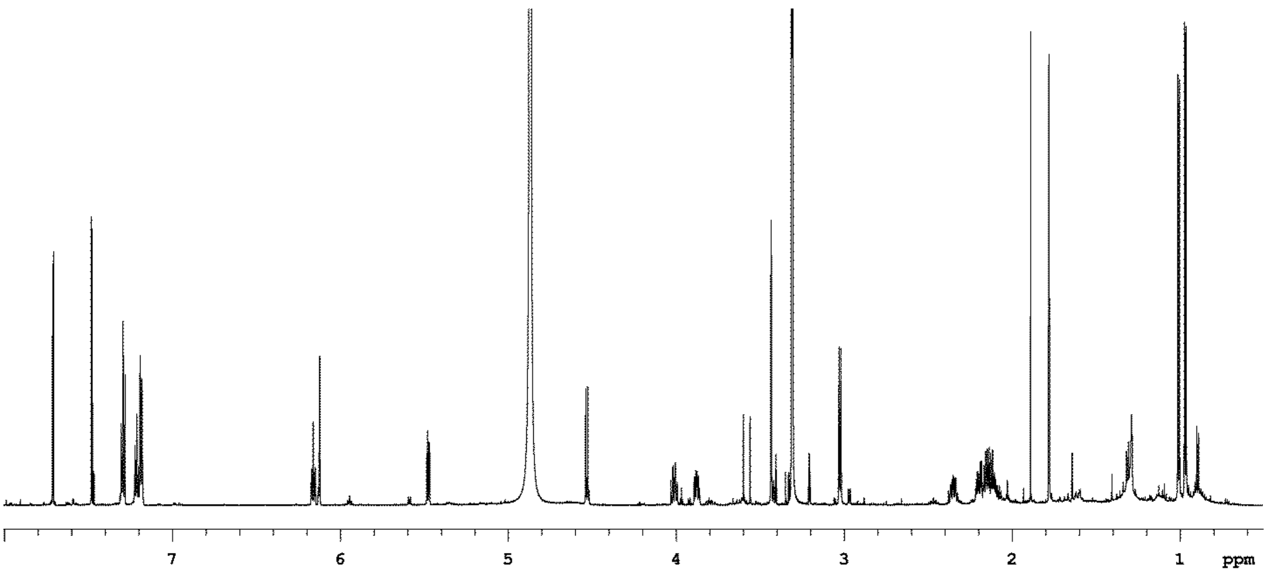

Figure S3. ^{1}H NMR spectrum of smenothiazole A (**3**) (CD_3OD, 700 MHz).

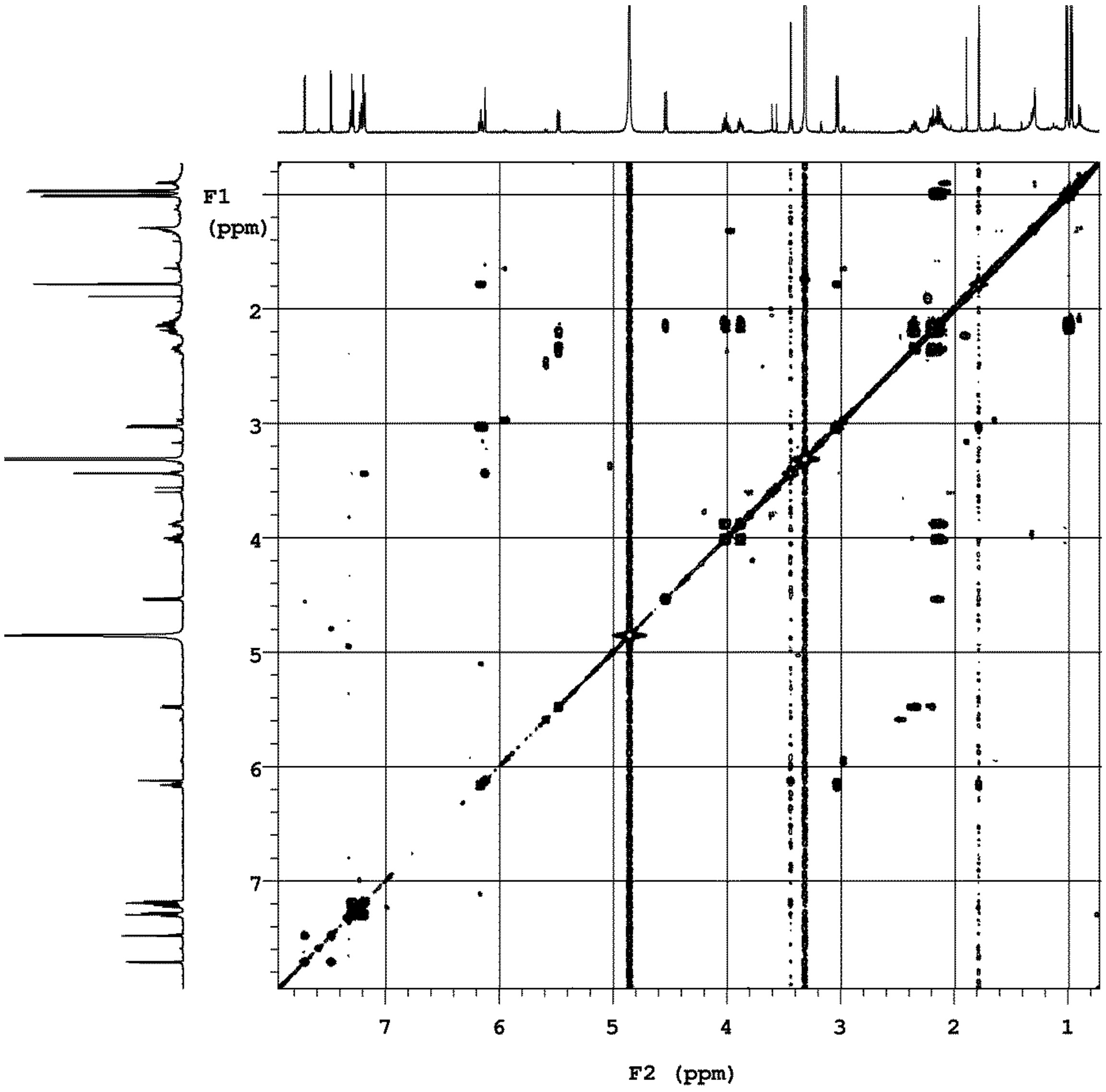

Figure S4. COSY spectrum of smenothiazole A (**3**) (CD_3OD, 700 MHz).

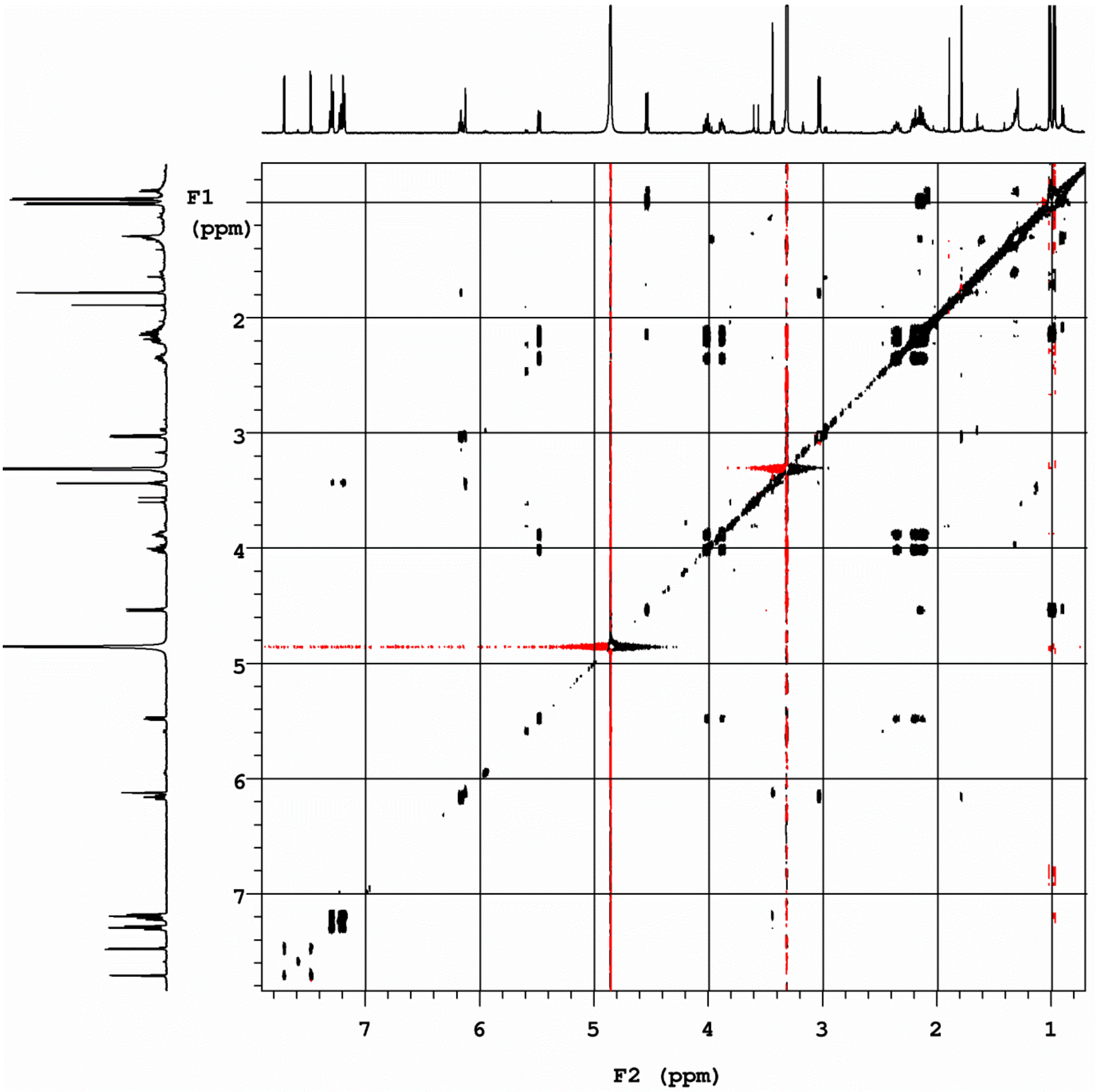

Figure S5. TOCSY spectrum of smenothiazole A (**3**) (CD_3OD, 700 MHz).

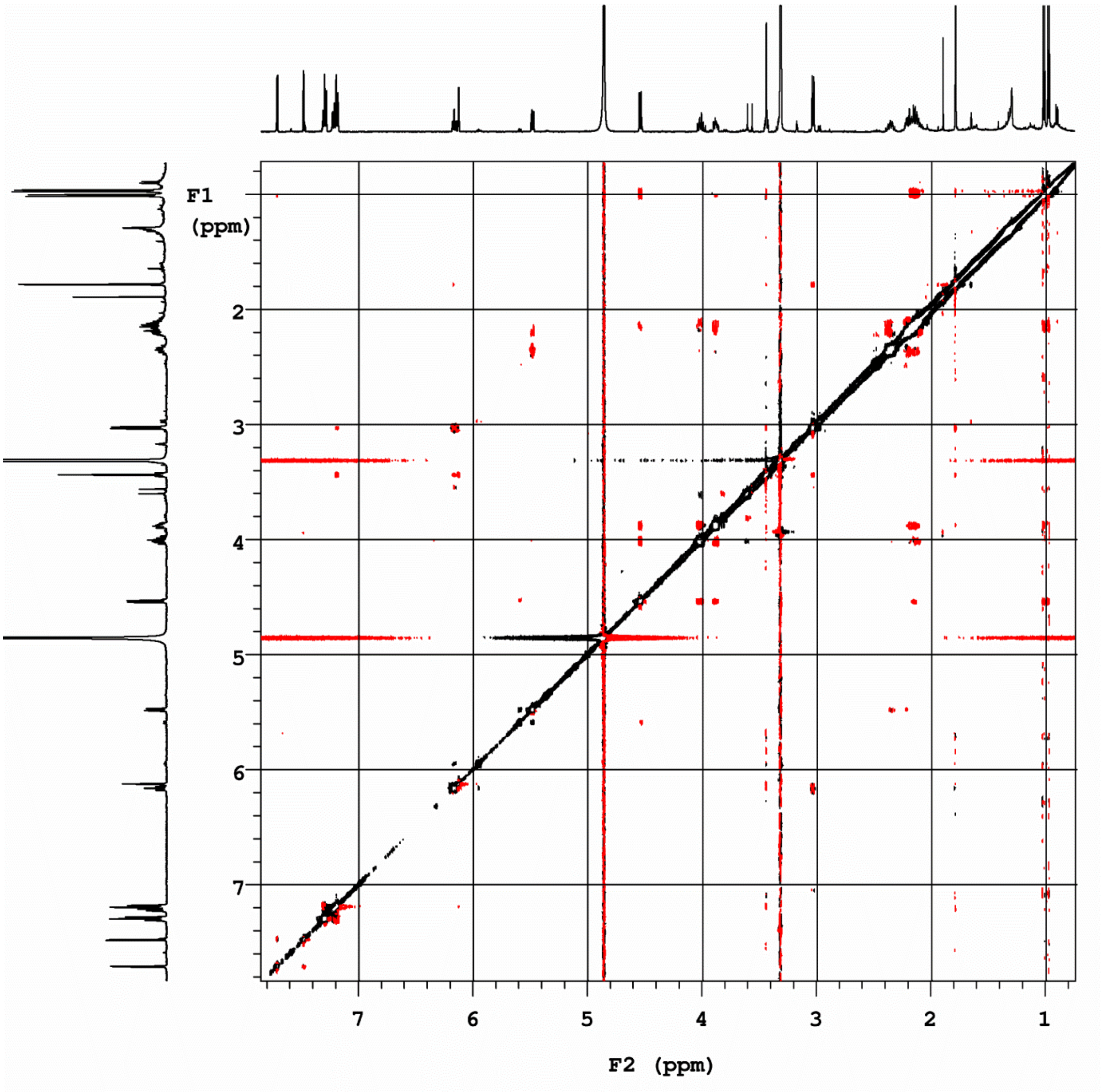

Figure S6. NOESY spectrum of smenothiazole A (**3**) (CD_3OD, 700 MHz).

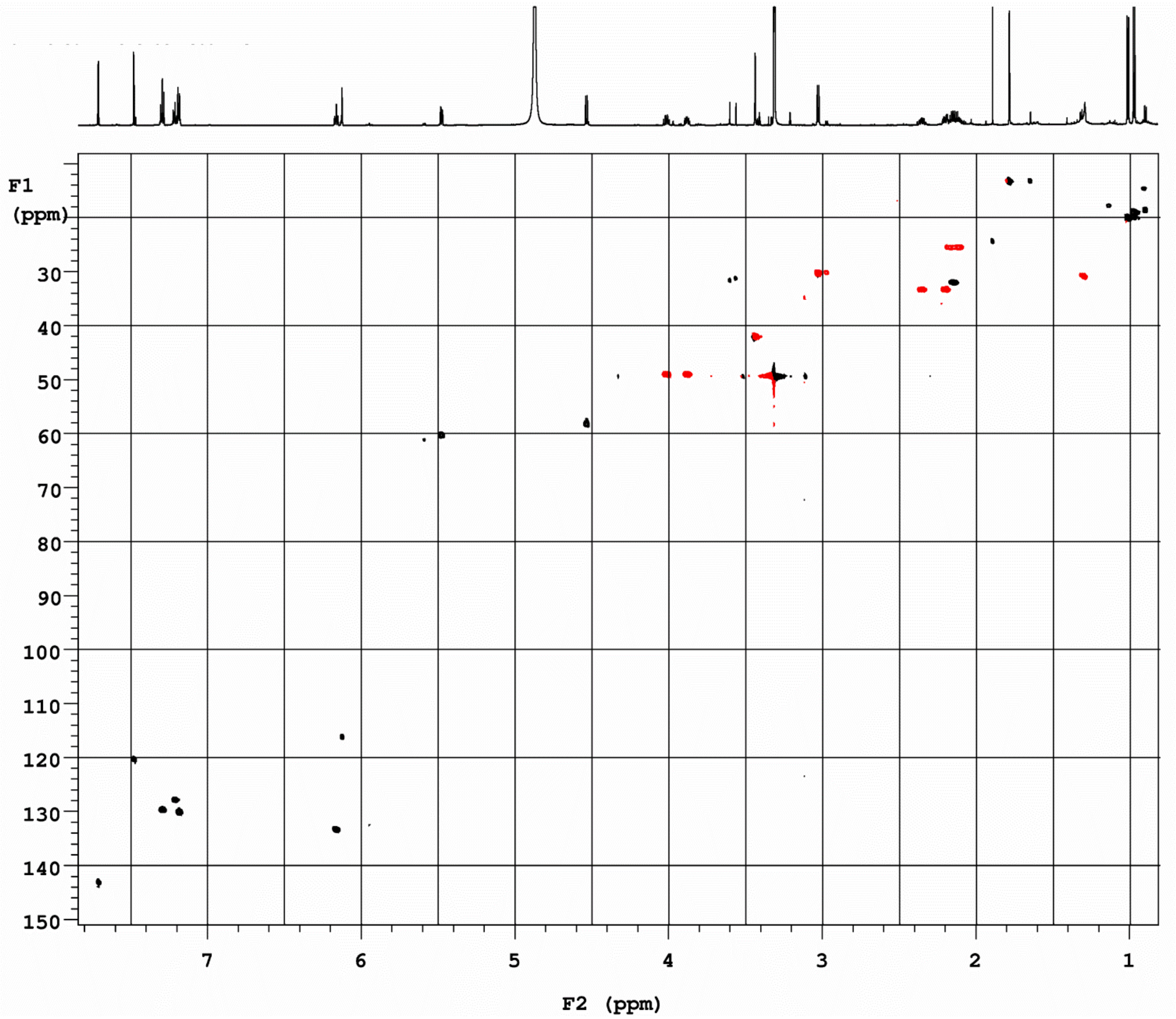

Figure S7. HSQC spectrum of smenothiazole A (**3**) (CD_3OD, 700 MHz).

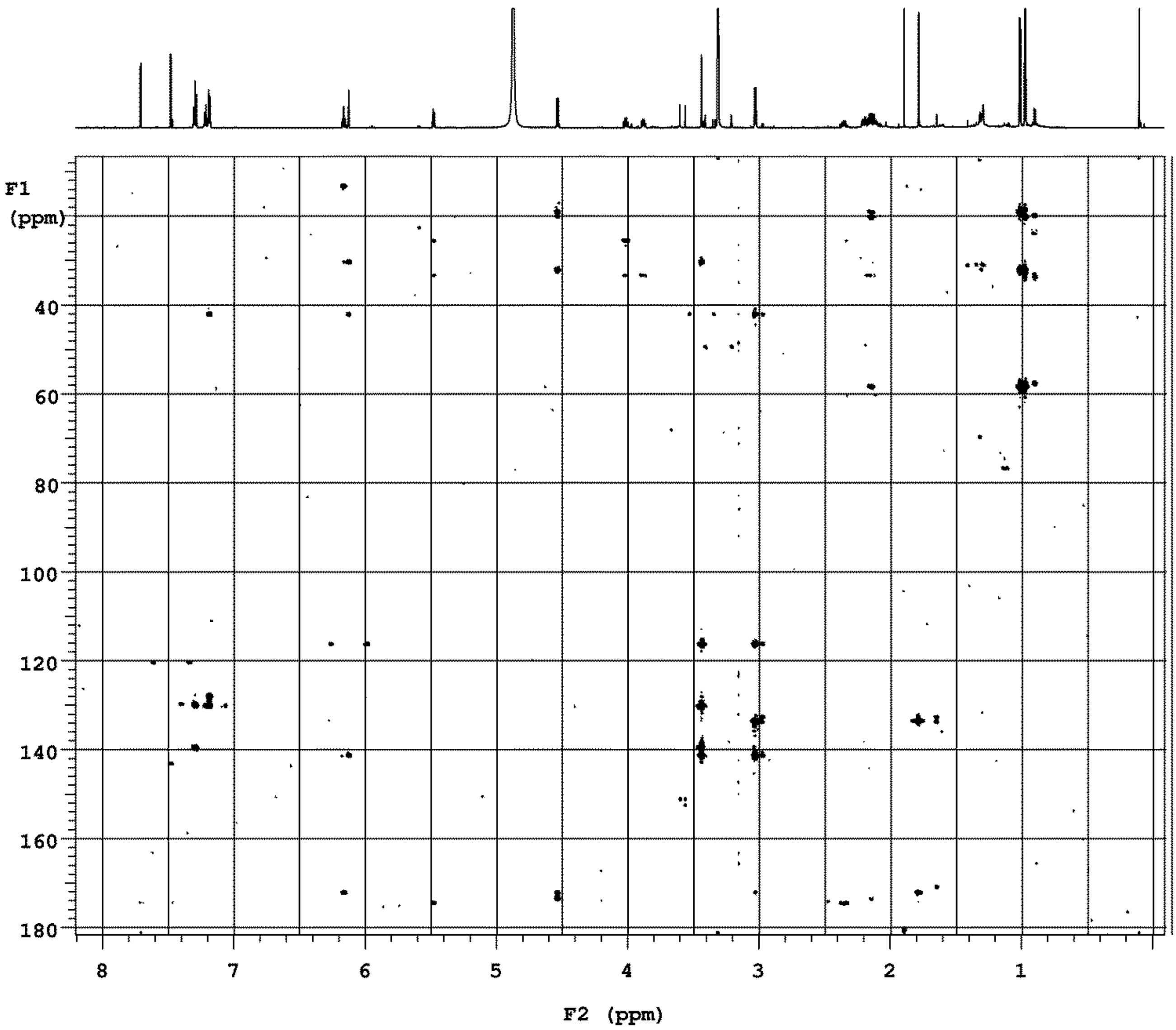

Figure S8. HMBC spectrum of smenothiazole A (**3**) (CD_3OD, 700 MHz).

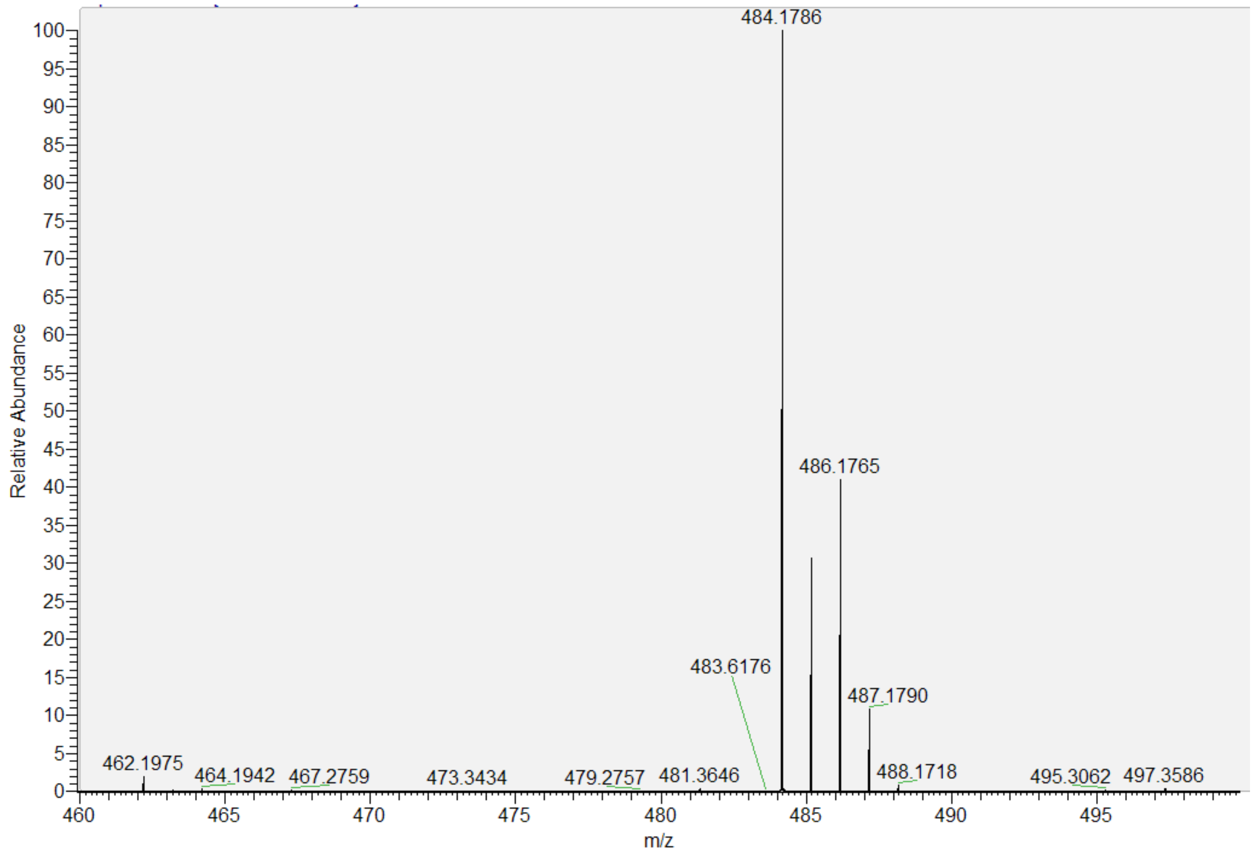

Figure S9. Positive ion mode high-resolution ESI MS spectrum of smenothiazole B (**4**).

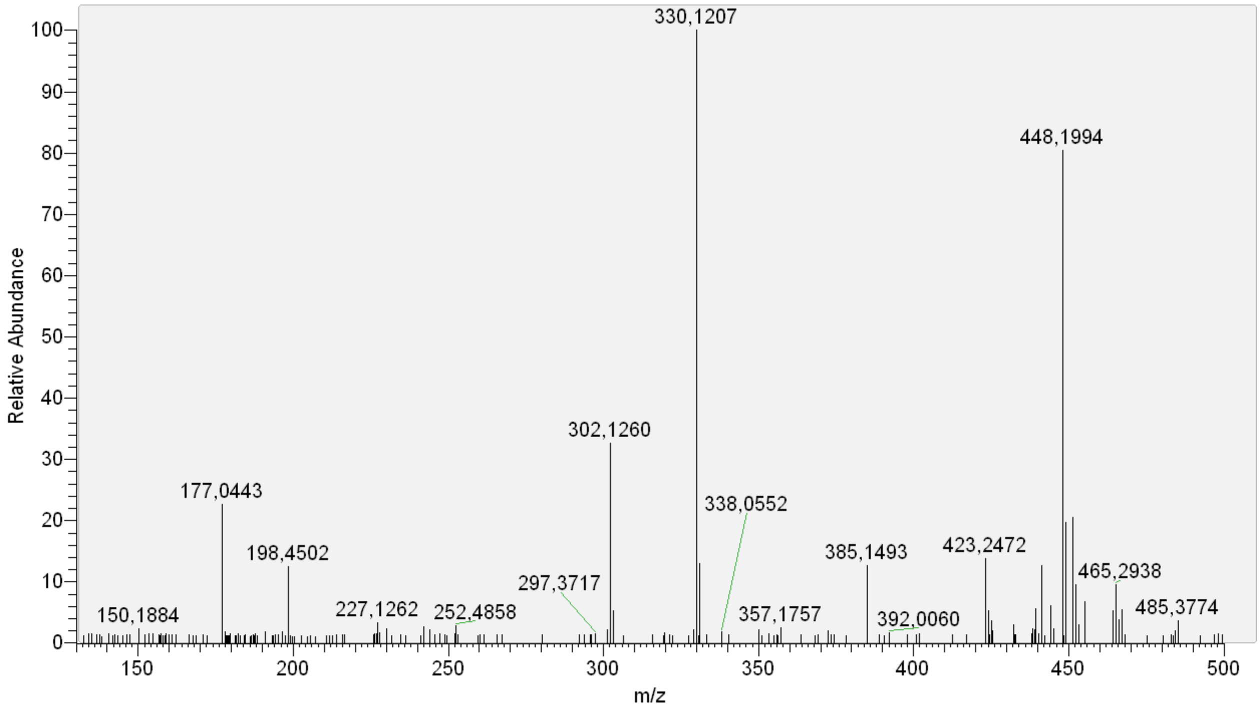

Figure S10. Positive-ion high-resolution ESI MS/MS spectrum of smenothiazole B (**4**), parent ion at *m/z* 484.18.

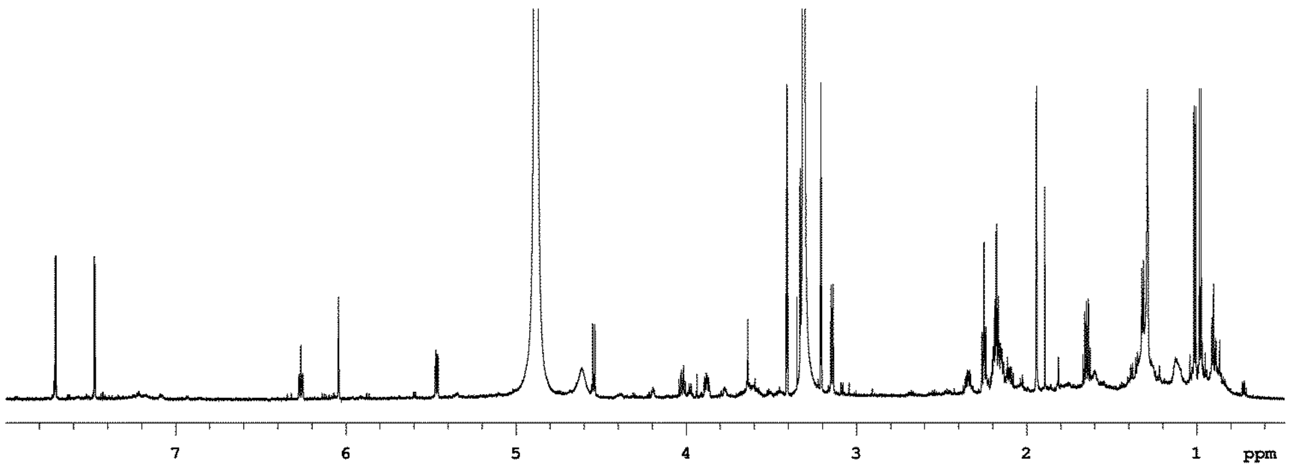

Figure S11. ^{1}H NMR spectrum of smenothiazole B (**4**) (CD_3OD, 700 MHz).

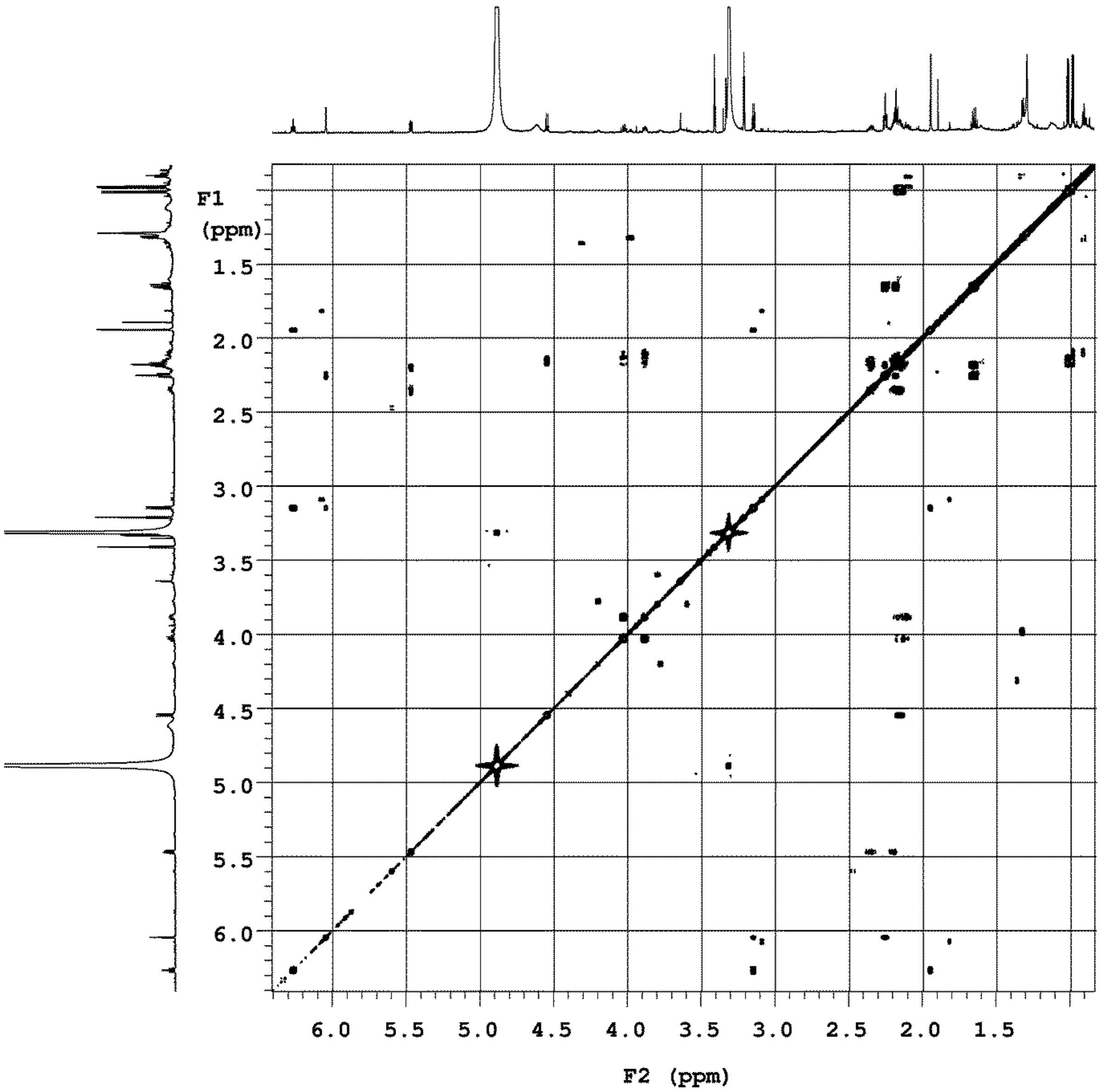

Figure S12. COSY spectrum of smenothiazole B (**4**) (CD_3OD, 700 MHz).

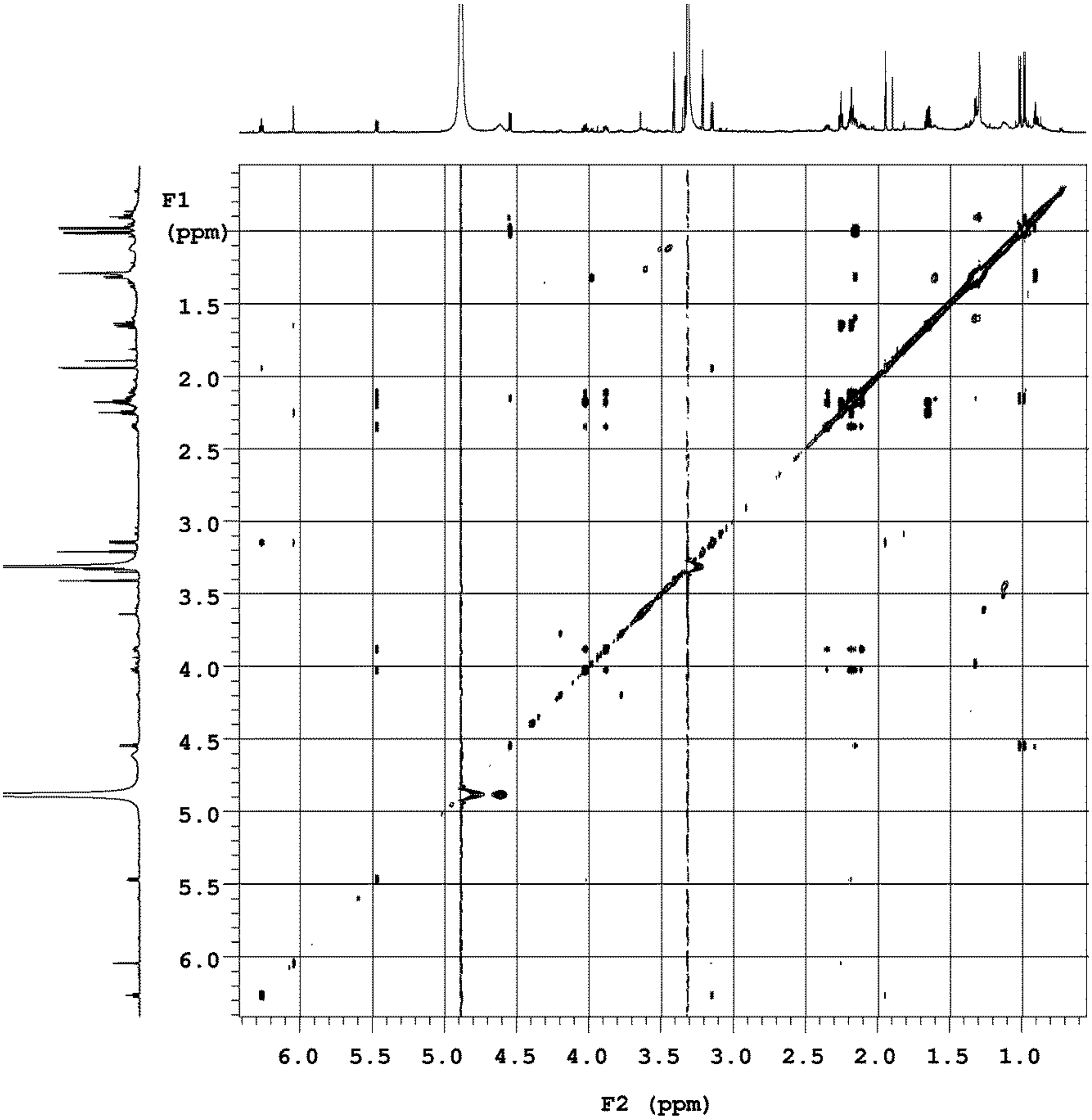

Figure S13. TOCSY spectrum of smenothiazole B (**4**) (CD_3OD, 700 MHz).

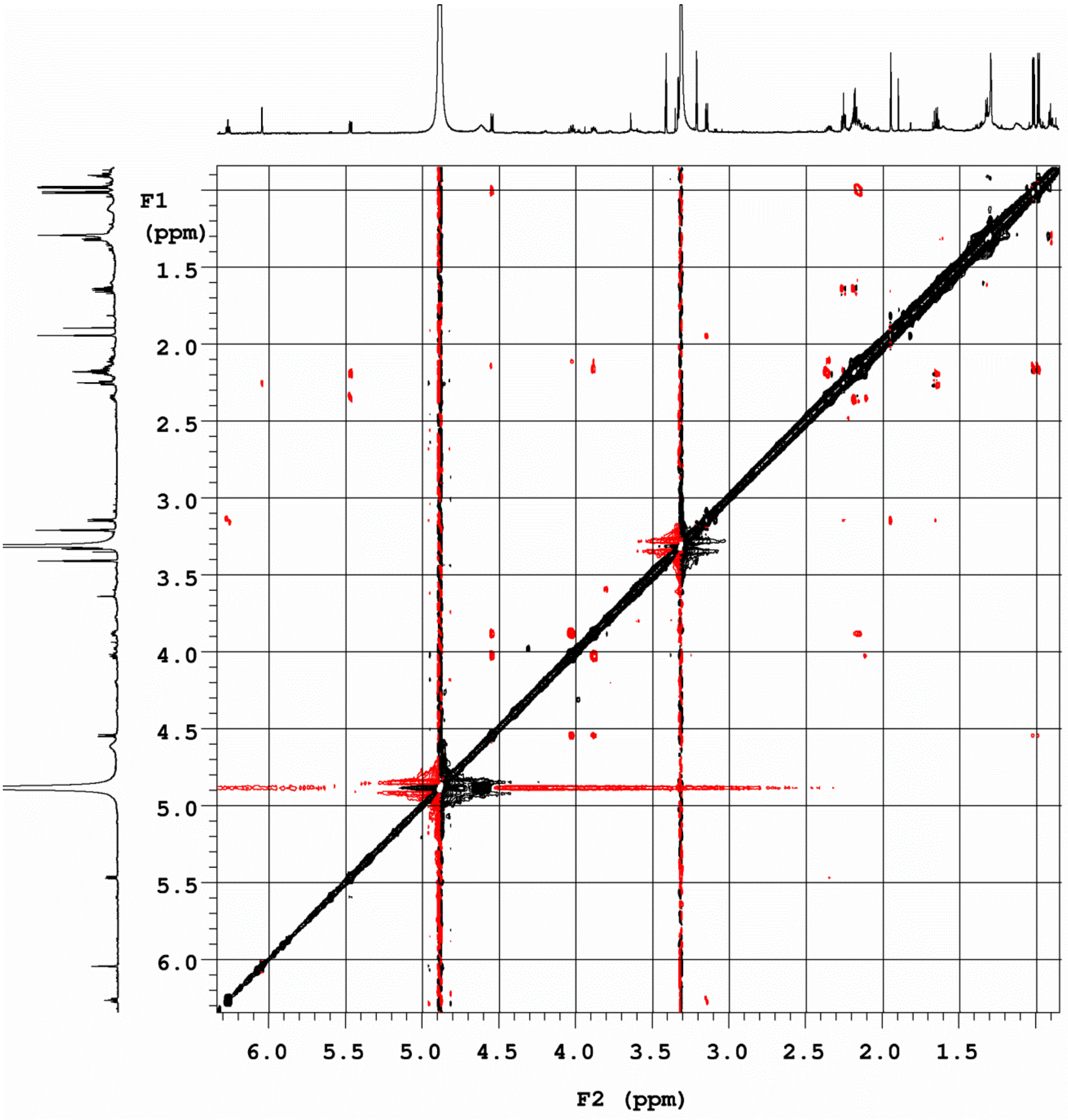

Figure S14. ROESY spectrum of smenothiazole B (**4**) (CD_3OD, 700 MHz).

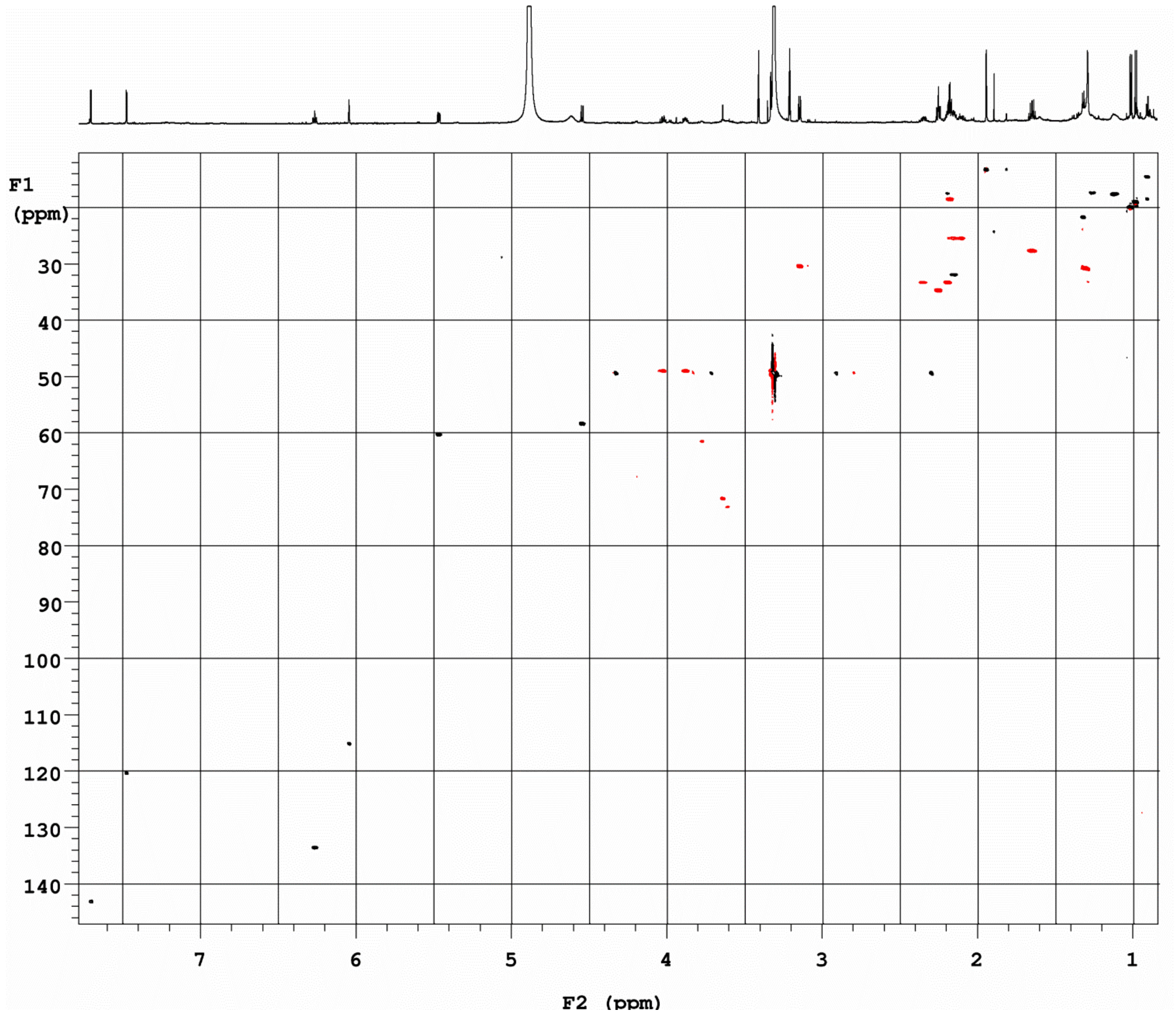

Figure S15. HSQC spectrum of smenothiazole B (**4**) optimized for $^{1}J_{CH}$ = 150 Hz (CD_3OD, 700 MHz).

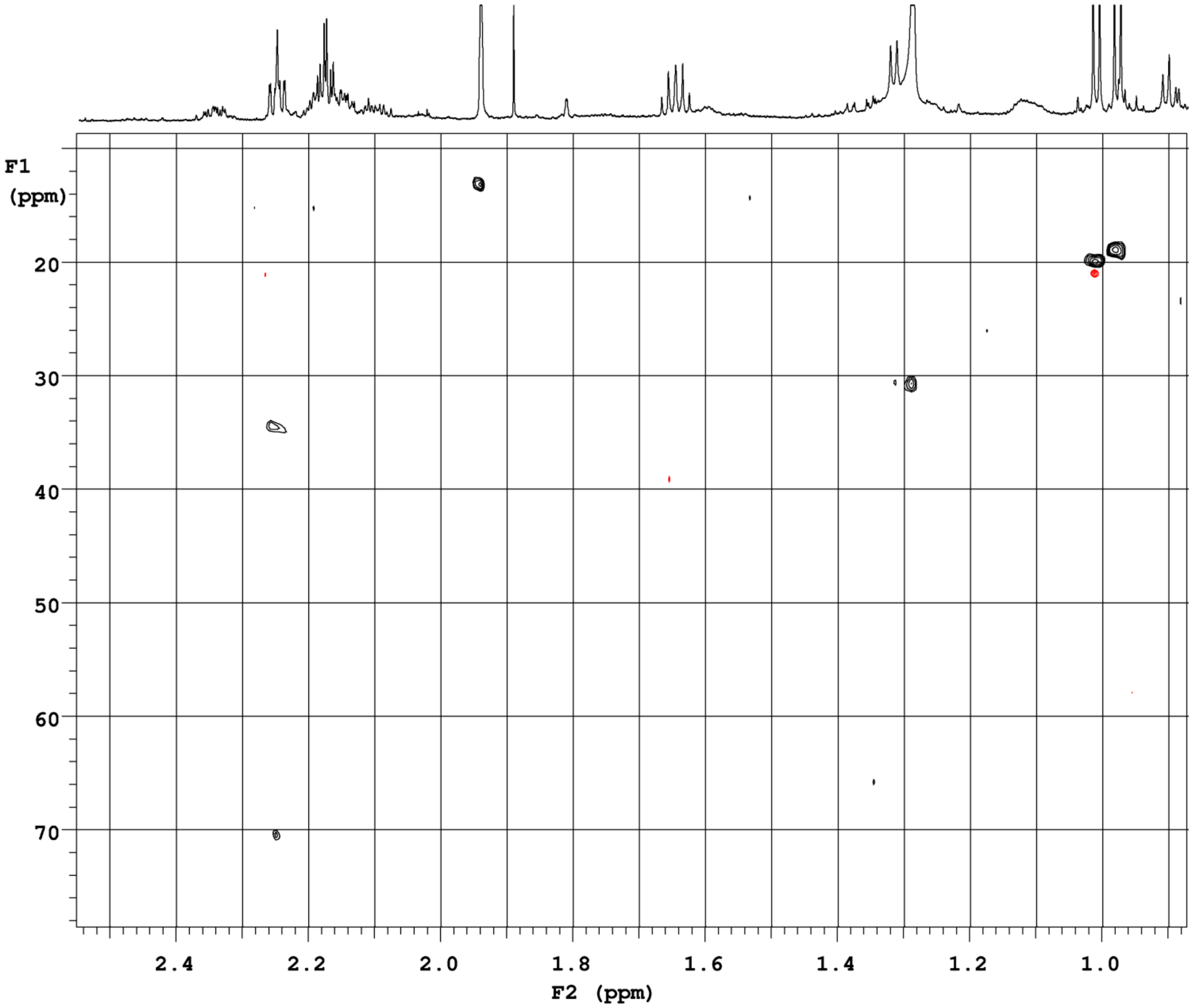

Figure S16. HSQC spectrum of smenothiazole B (**4**) optimized for $^{1}J_{CH}$ = 250 Hz (CD_3OD, 700 MHz).

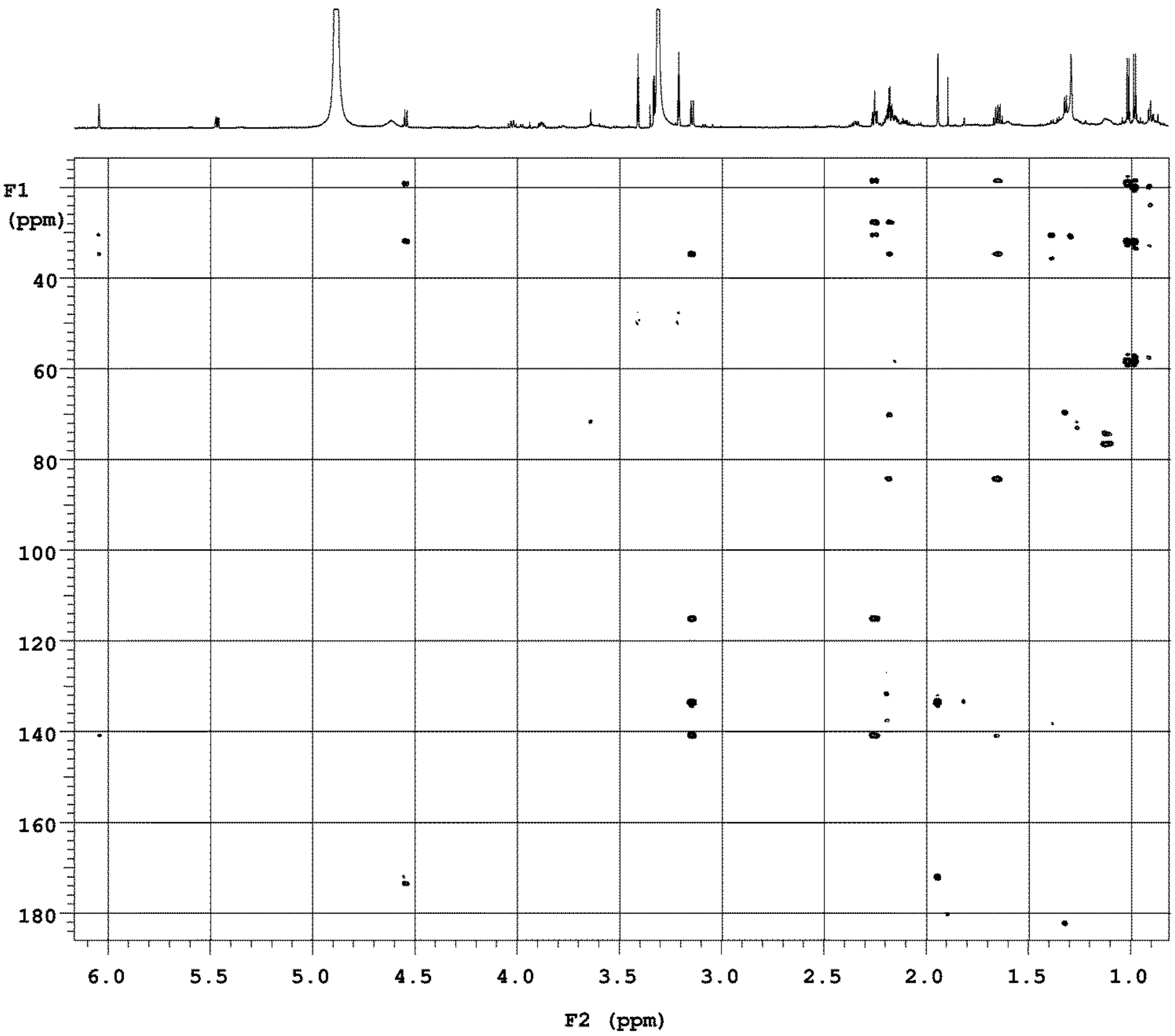

Figure S17. HMBC spectrum of smenothiazole B (**4**) (CD_3OD, 700 MHz).

© 2015 by the authors; licensee MDPI, Basel, Switzerland. This article is an open access article distributed under the terms and conditions of the Creative Commons Attribution license (http://creativecommons.org/licenses/by/4.0/).
